# Warning before misinformation exposure modulates memory encoding

**DOI:** 10.3758/s13415-024-01183-y

**Published:** 2024-03-19

**Authors:** Jessica M. Karanian, Ayanna K. Thomas, Elizabeth Race

**Affiliations:** 1 Department of Psychological and Brain Sciences, Fairfield University, 1073 North Benson Road, Fairfield, CT 06824 USA; 2 Department of Psychology, Tufts University, 419 Boston Ave, Medford, MA 02155 USA

**Keywords:** Misinformation effect, Prewarning, fMRI, Source misattribution, False memory, Prospective warning

## Abstract

Exposure to misleading information after witnessing an event can impair future memory reports about the event. This pervasive form of memory distortion, termed the misinformation effect, can be significantly reduced if individuals are warned about the reliability of post-event information before exposure to misleading information. The present fMRI study investigated whether such prewarnings improve subsequent memory accuracy by influencing encoding-related neural activity during exposure to misinformation. We employed a repeated retrieval misinformation paradigm in which participants watched a crime video (Witnessed Event), completed an initial test of memory, listened to a post-event auditory narrative that contained consistent, neutral, and misleading details (Post-Event Information), and then completed a final test of memory. At the behavioral level, participants who were given a prewarning before the Post-Event Information were less susceptible to misinformation on the final memory test compared with participants who were not given a warning (Karanian et al., *Proceedings of the National Academy of Sciences of the United States of America*, *117*, 22771–22779, 2020). This protection from misinformation was accompanied by greater activity in frontal regions associated with source encoding (lateral PFC) and conflict detection (ACC) during misleading trials as well as a more global reduction in activity in auditory cortex and semantic processing regions (left inferior frontal gyrus) across all trials (consistent, neutral, misleading) of the Post-Event Information narrative. Importantly, the strength of these warning-related activity modulations was associated with better protection from misinformation on the final memory test (improved memory accuracy on misleading trials). Together, these results suggest that warnings modulate encoding-related neural activity during exposure to misinformation to improve memory accuracy.

**Supplementary Information:**

The online version contains supplementary material available at 10.3758/s13415-024-01183-y.

## Introduction

Decades of research have identified *the misinformation effect*—a robust effect in which exposure to new information after an event can impair subsequent memory for the original event (for a review, see Loftus, 2005). To induce the misinformation effect, researchers present mock eyewitnesses with an event (i.e., Witnessed Event), often in the visual modality in the form of a video or a series of vignettes (Chan et al., 2009; Okado & Stark, 2005). After the event, mock eyewitnesses are provided with a verbal or visual recounting of the event (Post-Event Information). Critically, some of the Post-Event Information, referred to as misinformation, conflicts with the Witnessed Event. Later, when asked to answer questions about the Witnessed Event, mock eyewitnesses often falsely attribute the misinformation to the original witnessed event. The misinformation effect is traditionally measured by examining whether original event information is less accessible on a final test of memory and by examining the likelihood of producing or selecting the suggested misinformation in response to questions about the witnessed event.

Research has indicated that source misattribution may be one explanation for producing or selecting suggested misinformation in response to questions about the witnessed event. That is, when asked to retrieve an event detail, participants may retrieve a misleading detail and incorrectly attribute it to the original event (Ayers & Reder, 1998; Lindsay & Johnson, 1989a, 1989b). This consistent observation of the misinformation effect in the laboratory highlights the ease with which misleading information can be incorporated into memory and has implications for the reliability (or unreliability) of real-world eyewitness memory reports.

Importantly, a growing body of evidence suggests that providing a warning about the credibility of Post-Event Information can improve memory accuracy in the face of misinformation. For example, Blank & Launay (2014) conducted a meta-analysis and found that providing individuals with a retrospective warning immediately before a memory test (postwarning) cuts the size of the misinformation effect approximately in half (Blank & Launay, 2014). Participants were more likely to retrieve information from the witnessed event and less likely to produce or select misinformation. This protective effect of warning also has been observed in the context of repeated memory retrieval (Thomas et al., 2010), when participants recall details of an event before exposure to misinformation and susceptibility to misinformation is typically enhanced (Chan et al., 2009; Chan & LaPaglia, 2011; LaPaglia & Chan, 2013; Wilford et al., 2014; Gordon et al., 2015).

Postwarnings may encourage participants to consider the source or context in which each piece of information was first encountered (i.e., Witnessed Event vs. Post-Event Information) during the final memory test so that only information from the original Witnessed Event will be endorsed (Blank & Launay, 2014; for a review, see Johnson et al., 1993). Support for this proposal comes from a recent fMRI study by Karanian & colleagues (Karanian et al., 2020). In this study, participants watched a silent crime video (Witnessed Event), took an initial memory test about the video, listened to an audio narrative with misinformation (Post-Event Information), and then took a final memory test (Final Memory Test). When participants received a warning about the reliability of the Post-Event Information (either before or after the Post-Event Information), they were less likely to endorse misleading details as being from the original witnessed event during the final test of memory compared with when they were not warned. Importantly, warnings also influenced neural activity during memory retrieval, both by increasing activity in visual regions associated with the original source of information (video; Witnessed Event) as well as by decreasing activity in auditory regions associated with the misleading source of information (audio narrative; Post-Event Information). Stronger visual reactivation during memory retrieval was associated with reduced selection of misinformation, whereas stronger auditory reactivation was associated with increased selection of misinformation. These results suggest that warnings modulate reconstructive processes at the time of memory retrieval (Final Memory Test) and improve memory accuracy by encouraging retrieval from the original source of information (e.g., video; Witnessed Event) while reducing retrieval from the misleading source of information (e.g., audio narrative; Post-Event Information).

While the results of Karanian & colleagues (Karanian et al., 2020) highlight the impact of warnings on memory retrieval, prospective warnings given *before* Post-Event Information (prewarnings) also may impact the initial encoding of misinformation. To date, only a few studies have investigated the underlying mechanisms by which prewarnings influence memory accuracy in the face of misinformation. Greene et al. (1982) presented participants with a series of images depicting a burglary (Witnessed Event), had participants read a paragraph containing misleading information (Post-Event Information), and then administered a memory test (Final Memory Test). Participants who were warned about possible inaccuracies of the paragraph *before* exposure to the Post-Event Information committed fewer misinformation errors on the final memory test compared with unwarned participants. Critically, participants who received a prewarning also spent more time reading the Post-Event Information. These results suggest that in addition to influencing information processing at the time of memory retrieval, prewarnings also may improve memory accuracy by influencing encoding-related processes during exposure to misinformation (Gallo et al., 2001).

In line with this proposal, previous neuroimaging research suggests that encoding-related processes during exposure to misinformation play an important role in determining later memory accuracy in misinformation paradigms. For example, Okado & Stark (2005) found that during exposure to visual misinformation, activity in multiple brain regions, such as the prefrontal cortex, anterior cingulate cortex (ACC), and subregions of the medial temporal lobes (MTL), predicted later memory performance. Specifically, activity was greater for information that was subsequently remembered accurately as part of the original witnessed event compared with information that was subsequently misremembered (misleading information later inaccurately attributed to the original event). The authors suggested that this increased activity during exposure to misinformation might reflect enhanced source encoding, particularly when participants notice discrepancies with information presented in the original event phase, which reduces source misattribution during the final memory test. Indeed, previous behavioral work has indicated that detecting discrepancies or changes between an original Witnessed Event and Post-Event Information can reduce the misinformation effect (Tousignant et al., 1986; Putnam et al., 2017). By this logic, prewarnings could protect memory from misinformation by “tagging” discrepant misleading information with the source or context in which it was presented (enhanced source encoding).

Prewarnings also could have more global effects on how Post-Event Information is processed: for example, by reducing attention or by causing shallower processing of this information. Support for this proposal comes from a previous fMRI study by Baym & Gonsalves (2010), which investigated neural activity during exposure to verbal misinformation about a previous event. In this study, activity in default mode regions (e.g., precuneus/cuneus, anterior/posterior cingulate) during exposure to Post-Event Information was associated with accurate memory decisions in later tests of memory. Thus, prewarnings could have multiple potential effects on the processing of Post-Event Information.

### Current Study

While neuroimaging evidence is limited, the previous misinformation literature suggests that encoding-related processes during exposure to Post-Event Information influence subsequent memory performance. An important outstanding question is whether, and how, warnings modulate these encoding-related processes to improve memory accuracy. To investigate this question, the current study assessed the effect of prewarnings on neural activity during the Post-Event Information phase in the fMRI study previously reported by Karanian & colleagues (Karanian et al., 2020). Whereas the previous fMRI analyses presented by Karanian & colleagues (Karanian et al., 2020) exclusively focused on neural activity during memory retrieval (Final Memory Test), the present study investigates neural activity during exposure to misleading information (the Post-Event Information phase). In this paradigm, participants watched a silent crime video (Witnessed Event), took an initial memory test about the video, and then listened to a narrative that contained misleading details, consistent details, and neutral details (Post-Event Information). Finally, participants took a Final Memory Test, which asked them to remember details about the original Witnessed Event. Participants either were warned about the reliability of the Post-Event Information before exposure to it (prewarning), after exposure to it (postwarning), or did not receive a warning (no warning). The current study focused exclusively on data from the prewarning and no warning conditions given our interest in investigating the effect of warning on neural processing during the Post-Event Information and the relationship between such neural processing and performance on the Final Memory Test. Because a warning is administered *after* the Post-Event Information phase in the postwarning condition, it is problematic to make any inferences about the relationship between neural processing during the Post-Event Information and the Final Memory Test.

#### Hypotheses

If prewarnings modulate processing of Post-Event Information, then we would expect to find neural activity differences between the prewarning and no warning groups during the Post-Event Information phase. Based on the previous literature, we had two primary hypotheses. We first posited that prewarnings might encourage encoding of the context/source of information presented during the Post-Event Information, particularly when that information has been changed or conflicts with information presented in the original event (i.e., misleading information) (Green et al., 1982; Okado & Stark, 2005). According to this “enhanced source encoding” hypothesis, warned participants might be more likely to tag (or encode) misleading Post-Event Information with contextual or source information, which would help them to later disambiguate between information that came from accurate (Witnessed Event) versus inaccurate (Post-Event Information) sources during the Final Memory Test. If this is the case, then prewarnings should *increase* activity in regions that have been associated with detecting conflict (e.g., ACC; Carter & Van Veen, 2007) as well as regions associated with the encoding of contextual or source information into memory (e.g., inferior prefrontal cortex and MTL; Cansino et al., 2002; Krebs et al., 2015; for reviews, see Paller & Wagner, 2002; Kim, 2011). Second, we hypothesized that prewarnings could also improve memory accuracy by reducing processing of the Post-Event Information at a more global level—for example, as a result of not paying as much attention to that information (Baym & Gonsalves, 2010). According to this “attenuated processing” hypothesis, reduced attention to and processing of the Post-Event Information could lead to a less robust memory representation of the Post-Event Information and, in turn, reduce the likelihood of retrieving misleading details on the final memory test. If this is the case, the prewarning group should demonstrate decreased activity across trials in the Post-Event Information phase in brain regions associated with sensory perceptual (i.e., auditory cortex) or attentional processing (e.g., frontoparietal network). Attenuated processing of the Post-Event Information in the prewarning group also could be associated with increased activity in default mode regions (Baym & Gonsalves, 2010).

## Materials and methods

### Participants

Eighty adult participants aged 18–35 years were recruited from the Boston area and were compensated $20/hour for participation. All participants were right-handed, native English speakers, had normal or corrected-to-normal vision, and reported no history of traumatic head injury. Fifteen participants were excluded in total: 11 participants were excluded before fMRI analysis because of technical problems during scanning, three participants were excluded because of noncompliance during the scanning session (e.g., falling asleep), and one participant was excluded for being outside the target age range (aged >35 years). This yielded a final sample of 65 participants (No Warning: *n* = 22, Prewarning: *n* = 21, Postwarning, *n* = 22; *M*_age_ = 24, *SD* = 4; 57% female). However, only individuals in the No Warning (*n* = 22) and Prewarning (*n* = 21) groups were included in the present analyses given the scope of this paper. All participants provided informed consent in accordance with the procedures of the institutional review board at Tufts University.

### Stimuli

#### Witnessed Event

During the encoding period, participants viewed a 22-min video clip from the black and white silent film *Rififi* (Bezard et al., 1955). The video depicts the events surrounding a burglary of a jewelry store and contains no dialogue.

#### Initial Memory Test

Participants’ memory for the 24 critical details from the witnessed event (Witnessed Event) was assessed in a four alternative forced choice recognition memory test. The 24 questions were presented, one at a time, and each asked about a critical detail from the witnessed event. Critical details were probed in chronological order (e.g., the same order in which they appeared in the video). Four alternative answers were displayed below each question and consisted of the correct detail shown in the video, a misleading detail, and two highly plausible lures (as determined by pilot testing). The order of answers was randomized across participants. Each question was displayed for 7 s, during which participants made their memory decisions, followed by a 500-ms blank screen. Then, participants were given 3 s to indicate their level of confidence via on a ordinal scale that ranged from 1–4 with 1 representing guess/low confidence and 4 representing high confidence. A jittered interstimulus interval with an average of 8 s was inserted between each memory question. Participants made a left/right button press, indicating the direction of a series of arrows. For the initial memory test, the test was presented on a laptop and all button presses were made on the keyboard (Karanian et al., 2020).

#### Post Event Information

An auditory narrative synopsis of the witnessed event was recorded by a female speaker (Karanian et al., 2020). Twenty-four sentences contained critical details that would be probed during the memory tests. These sentences either (1) accurately described a detail from the witnessed event (consistent) (e.g., “A rope is secured to an umbrella”), (2) inaccurately described a detail from the witnessed event (misleading) (e.g., “A rope is secured to a bucket”), or (3) provided an alternative (neither consistent nor inconsistent) detail from the witnessed event (neutral) (e.g., “A rope is secured to an object”). Critical details always appeared at the end of the sentence. An equal number of critical sentences contained consistent, neutral, or misleading details. Critical sentences were separated by at least three filler sentences that were not probed in the subsequent tests of memory. Each critical detail appeared only once during the narrative and the assignment of each detail to the consistent, neutral, or misleading condition was counterbalanced across participants. A jittered interstimulus interval with an average of 6 s (range 4–8 s) was inserted between each sentence during which a series of arrows pointing left or right was presented. Participants made a left/right button press indicating the direction of the arrows on a button box. In addition, because of a coding error, one sentence near the end of the narrative appeared three times (once in each condition) for a subset of participants (*n* = 24). Behavioral analyses showed no differences in response patterns between participants who did and did not hear this erroneous item. Thus, we have no reason to believe that it had a meaningful effect on the present data. Nonetheless, this item was omitted from the behavioral and imaging data. 

#### Final Memory Test

The content of the final memory test was identical to that described above for the initial memory test. However, the final memory test was completed while participants were in the fMRI scanner. Accordingly, the final memory test was presented on the in-scanner projector screen and all responses were made on a MRI-compatible button box (Karanian et al., 2020).

### Procedure

The experiment consisted of four stages: a video of a witnessed event, an initial memory test, an audio narrative recounting the witnessed event that included misleading details, and a final memory test (Karanian et al., 2020). Participants watched the video clip of the witnessed event on a computer monitor outside the scanner. Immediately following the video, participants were administered the first memory test on a laptop computer before entering the MRI scanner. Then, participants entered the scanner and both the audio narrative and the final memory test occurred while brain images were acquired. Participants listened to the audio narrative through MRI-compatible earphones and viewed the final memory test questions on an overhead mirror that contained the image of a screen onto which the questions were projected. Participants were instructed to minimize movement as much as possible during scanning and indicated their responses on a button box. Participants were randomly assigned to no warning, prewarning (warned before the audio narrative), or postwarning (warned after the audio narrative) conditions. As described earlier, the primary manipulation of interest in the present study was whether participants received a warning about the veracity of the Post-Event Information before it was presented (prewarning) or not at all (no warning).

Before the Post-Event Information, participants in the prewarning condition were provided the following instructions: *“You will have to answer questions regarding the video you previously watched for a second time. We will play a narrative of that video; however, we are uncertain as to the source of the narrative. Therefore, we were unable to verify the accuracy of the narrative. As such, base your answers only on what you saw in the video, and not on what you hear in the narrative. During the narrative, please keep your eyes fixed on the cross on the screen.”* Alternatively, participants in the no warning condition were provided the following instructions before the Post-Event Information: *"You will now hear an audio narrative of the video you just watched. During the narrative, please keep your eyes fixed on the cross on the screen.”* Both the no warning and prewarning conditions were provided the following instructions before the Final Memory Test: *"You will now answer a series of questions relating to the video you watched at the beginning of this experiment. Please answer each question to the best of your ability. If you do not know the answer, make your best guess. All questions must be answered. Select your answer using the numbers and then wait for the screen to advance. After each question, please rate your confidence in your answer on a scale from 1, complete guess to 4, high confidence.”* Task instructions and warnings for all conditions can be found in Appendix A.

### Behavioral analysis

Behavioral results are a subset of those previously reported in Karanian et al. (2020), restricted to comparison of prewarning and no warning groups. Recognition memory performance on the initial and final memory tests was calculated by dividing the total number of trials in which participants selected a correct video detail by the total number of trials for that given item type (consistent, neutral, misleading). Misinformation selection (a term that can be used interchangeably with “misinformation endorsement”) was calculated as the proportion of misleading trials in which participants selected the misleading detail that had been inaccurately described in the auditory narrative. This terminology has been used in recent studies using misinformation paradigms with recognition tests (O’Donnell & Chan, 2023; Karanian et al., 2020). As a baseline comparison, we also counted the proportion of consistent and neutral trials in which participants spontaneously selected a misleading detail (that had not been mentioned in the auditory narrative).

### MRI data collection and preprocessing

Structural and functional images were acquired on a Siemens 3T Magnetom Prisma Fit scanner (Siemens Medical, Erlangen, Germany) with a 32-channel head coil at the MIT Athinoula A. Martinos Imaging Center. Functional data were acquired using a T2*-weighted echo-planar imaging sequence (TR = 2000 ms, TE = 30 ms, flip angle = 90º, field-of-view = 210 x 210 mm, matrix = 64 x 64, slice thickness = 3.0 mm). Forty axial slices parallel to the AC-PC line were obtained. High-resolution structural images of the whole brain were acquired by using a T1-weighted, rapid gradient echo pulse sequence (MPRAGE; TR = 1800 ms, TE = 2.36 ms, flip angle = 8º, field-of-view = 250 x 250 mm, slice thickness = 0.87 mm; 208 slices, 0.9- x 0.9- x 0.9-mm resolution; Karanian et al., 2020).

Image preprocessing and data analysis was performed by using SPM12 (Wellcome Department of Cognitive Neurology, London, UK). Functional volumes for each participant were slice-time corrected with the middle slice in time used as a reference and corrected for head motion. The T1-weighted anatomical volume was coregistered to the functional data and segmented into gray and white matter. Segmented images were used to calculate spatial normalization parameters to Montreal Neurological Institute (MNI) space, which were then applied to the functional data. As part of spatial normalization, data were resampled to 2 x 2 x 2 mm. Functional images were then spatially smoothed with a 4-mm FWHM Gaussian kernel (Karanian et al., 2020).

### fMRI analysis

Functional data from the Post-Event Information phase in which participants listened to the audio narrative were analyzed by using a general linear model (GLM), including separate regressors each of the following: consistent trials, neutral trials, misleading trials, arrows task (baseline), and six nuisance regressors for each of the motion correction parameters. Trials were modeled as epochs defined by the onsets and duration (4 s) of each sentence of the narrative and were convolved with the canonical hemodynamic response function. A high-pass filter of 1/128 Hz was applied to remove low-frequency noise. Contrasts of interest were computed for each participant at the single-subject level and subjected to a random effects (second-level) GLM analysis to investigate the effect of warning (prewarning vs. no warning). We first examined whether warnings increased neural activity during exposure to misinformation by comparing activity in the prewarning versus no warning group on misleading trials (Misleading – Arrows). Next, we examined whether warnings had a more global effect on processing across all trials during the Post-Event Information phase by comparing activity in the prewarning versus no warning group across all trials, which includes both the 24 critical trials as well as the filler sentences (All Trials – Arrows).

### fMRI statistical analysis

Whole-brain contrasts of interest were performed to investigate differences between the two warning conditions: prewarning and no warning. We employed an individual voxel threshold of *p* < 0.005, and Monte Carlo simulations were used to determine a cluster extent of *k* = 38 to correct for multiple comparisons to *p* < .05 (https://www2.bc.edu/sd-slotnick/scripts.htm; 1,000 iterations, full width at half maximum = 6 mm; Kark et al., 2020; Slotnick, 2023, Slotnick et al., 2003). We primarily focus on the activations that exceeded the corrected minimum cluster size of *k* = 38. However, for completeness and to minimize Type II error, we also report significant regions with a cluster size of *k* = 10 in the Supplemental Results.

Beta-weights were extracted from each cluster of activity and entered into 2 x 3 mixed ANOVAs to assess the effect of condition (prewarning, no warning) and trial type (consistent, neutral, misleading). Post hoc independent sample *t*-tests were conducted when warranted. Beta weights extracted from these activations were used to assess the relationship to behavior. Specifically, two-tailed Pearson correlations assessed the relationship between activation and later susceptibility to misinformation during the final memory task (i.e., memory accuracy on misleading trials).

We also conducted targeted analyses of neural activity in a priori regions of interest in the MTL, including the hippocampus and the parahippocampal cortex (Okado & Stark, 2005). Anatomical ROIs were drawn from the AAL anatomical atlas (Tzourio-Mazoyer et al., 2002) by using the WFU PickAtlas. Beta weights from these anatomical ROIs were then extracted from the contrasts of interest to assess whether there were differences based on warning condition. Two-tailed Pearson correlations were again conducted to assess the relationship between activation and memory accuracy on misleading trials on the final memory test.

## Results

### Behavioral results

Behavioral results have been previously reported by Karanian & colleagues (Karanian et al., 2020) but are summarized here to highlight comparison of the prewarning and no warning groups. Table 1 includes means and standard errors by condition and trial type for each dependent variable reported below (accuracy, misinformation selection, confidence, reaction time).

**Table 1:** Behavioral results. Average proportion correct, misinformation selection, confidence, and reaction time as a function of trial type (consistent, neutral, misleading) and warning condition (no warning, prewarning) during the final memory test

	Consistent	Neutral	Misleading
*No warning (N = 22)*			
Accuracy	0.82 (0.05)	0.63 (0.06)	0.37 (0.05)
Selection	0.04 (0.02)	0.18 (0.04)	0.52 (0.04)
Confidence	3.49 (0.10)	2.84 (0.07)	3.32 (0.08)
Reaction time	6152.54 (51.17)	6282.84 (52.09)	6160.09 (44.64)
*Prewarning (N = 21)*			
Accuracy	0.78 (0.05)	0.64 (0.05)	0.59 (0.05)
Selection	0.09 (0.02)	0.20 (0.03)	0.29 (0.05)
Confidence	3.27 (0.10)	2.93 (0.10)	3.14 (0.10)
Reaction time	6106.44 (48.75)	6175.41 (48.75)	6243.10 (48.75)

*Note:* Means and standard errors are reported

**Fig. 1:**
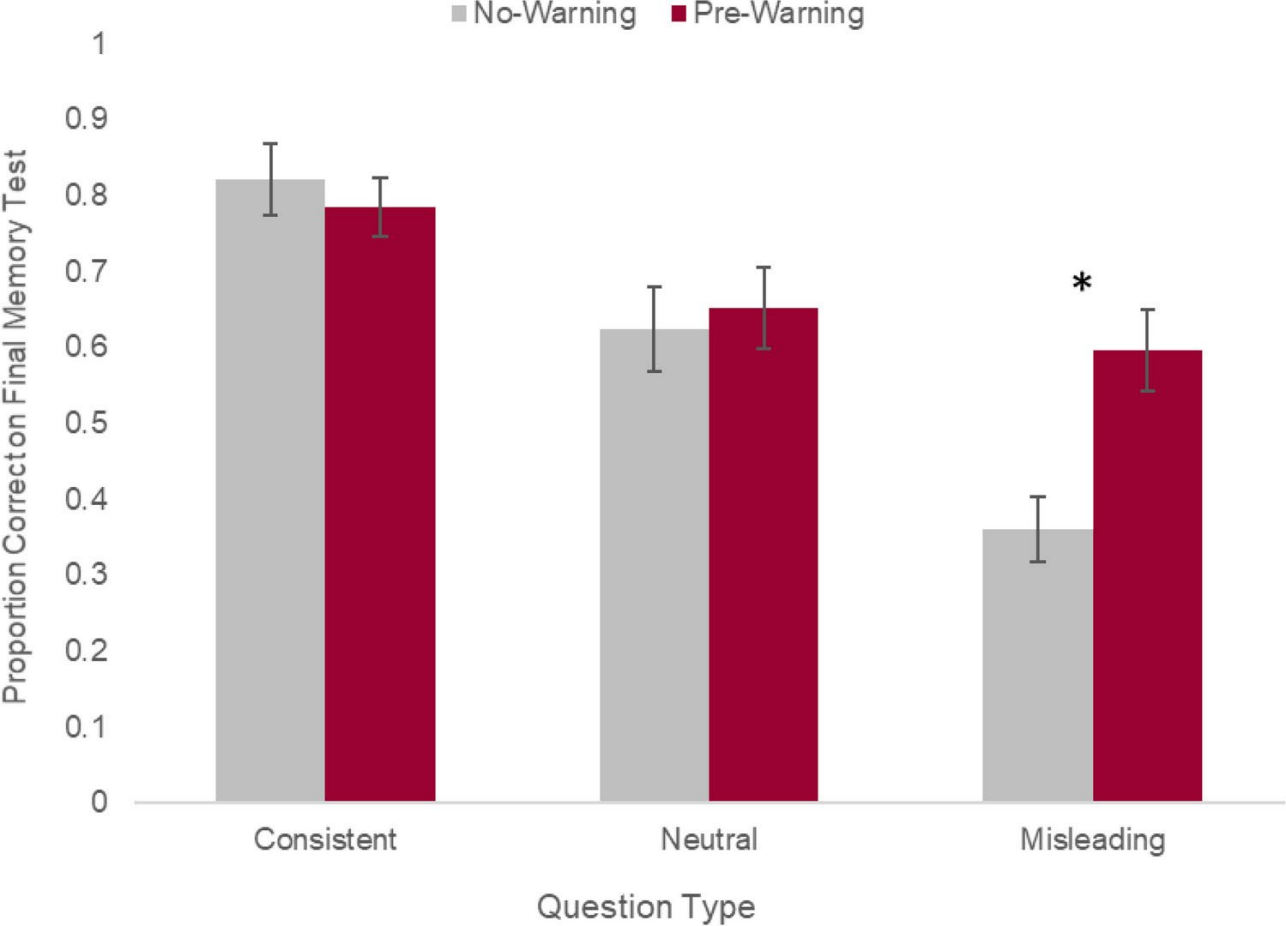
Behavioral results from the final memory test. Proportion correct refers to the proportion of trials within each trial type (consistent, neutral, misleading) that were answered correctly (i.e., the number of trials in which participants selected the correct video detail divided by the total number of trials within that trial type). Error bars indicate between-participant SEs. *p < 0.001

On the final memory test, a strong misinformation effect was observed across all participants such that accuracy was reduced for misleading trials (*M* = .48), relative to neutral trials (*M* = .63) and consistent trials (*M* = .80; *F*(2, 82) = 32.95, *p* < .001, *n*^*2*^_*p*_ = .45; Fig. 1). Importantly, there was a significant interaction between trial type (consistent, neutral, misleading) and warning condition (no warning, prewarning) (*F*(2, 82) = 6.09, *p* < .005, *n*^*2*^_*p*_ = .13), whereby prewarnings improved memory accuracy on misleading trials (*t*(41) = 3.12, *p* < .003, *d* = .95) at no cost to memory accuracy on consistent trials (*t*(41) < 1) or neutral trials (*t*(41) *< 1*).

Prewarning similarly reduced misinformation selection across all trials during the final memory test (*F*(2, 82) = 4.86 , *p* = .03, *n*^*2*^_*p*_ = 0.2). More specifically, prewarning reduced the likelihood of selecting misinformation on misleading trails (*t*(41) = 3.83, *p* < .001, *d* = 1.17) but did not affect the likelihood of selecting misinformation on consistent (*t*(41) = 1.7, *p* > .05) or neutral (*t*(41) > 1) trials during the final memory test. As anticipated, accuracy on misleading trials and misinformation selection showed a strong negative correlation (*r* = −0.79, *p* < .001, 95% confidence interval [CI] [−0.64, −0.88]).

There was no effect of warning or trial type on reaction time during the final memory test (*F*(2, 82) = 2.25, *p* > .05). Although there was an interaction between warning and trial type on confidence ratings (*F*(2,82) = 3.19, *p* < .05,* n*^*2*^_*p*_ = .07), post-hoc *t*-tests revealed no differences in confidence between groups on consistent (*t*(41) = 1.59, *p* > .05), misleading (*t*(41) = 1.44, *p* > .05), or neutral trials (*t*(41) < 1). As anticipated, there was a significant main effect of trial type (*F*(2, 82) = 29.24, *p* < .001,* n*^*2*^_*p*_ = .42) such that confidence was significantly higher on consistent trials (*M* = 3.38) compared with misleading trials (*M* = 3.23, *t*(41) = 2.24, *p* < .05, *d* = .34) and neutral trials (*M* = 2.88, *t*(41) = 7.45, *p* < .001, *d* = 1.14), and confidence on misleading trials was significant greater than neutral trials (*t*(41) = 5.21, *p* < .001, *d* = .80).

### fMRI results

#### Enhanced Source Encoding Hypothesis

**Fig. 2:**
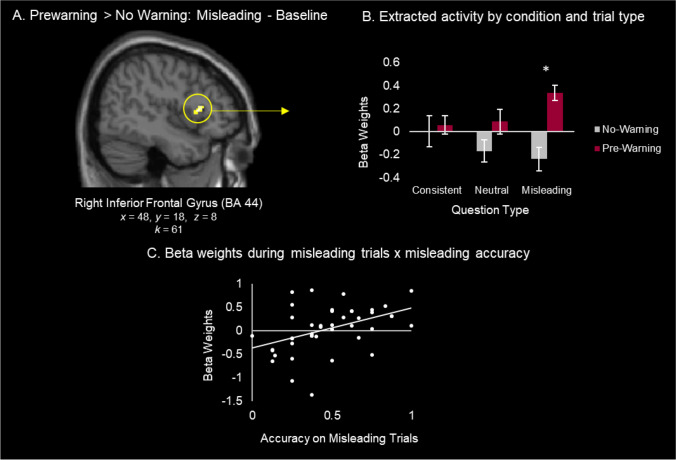
Whole Brain: Prewarning > No Warning. (**A**) Frontal activation was revealed by the contrast of prewarning > no warning for Misleading Trials – Baseline. (**B**) Extracted beta weights are plotted by trial type and condition. (**C**) Collapsing across warning condition, extracted beta weights are plotted in relation to accuracy on misleading trials on the final memory test

We first examined whether prewarnings increase encoding-related neural activity during exposure to misinformation by comparing activity in the prewarning versus the no warning groups on misleading trials. Activity was greater in the prewarning compared with the no warning group in the right inferior frontal gyrus (IFG; BA 44; *x* = 48, *y* = 18, *z* = 8) (Fig. 2A). Follow-up analysis revealed that this activity difference between groups was specific to the misleading trails (Fig. 2B). Specifically, parameter estimates extracted from this region were entered into a condition (prewarning, no warning) by trial type (consistent, neutral, misleading) repeated-measures ANOVA (Fig. 2B), revealing a significant effect of condition (*F*(1,41) = 9.89, *p* = .003, *n*^2^_p_ = .19) and a significant interaction between condition and trial type (*F*(2, 82) = 3.91, *p* = .024, *n*^*2*^_*p*_ = .09), with no effect of trial type (*F* < 1). Post hoc independent samples *t*-test revealed that this pattern was driven by a significant increase in activity on misleading trials in the prewarning group (*M* = 0.34) compared with the no warning group (*M* = −0.24; *t*(41) = 4.71, *p* < .001, *d* = 1.44). There were no significant differences between groups on neutral trials (*t*(41) = 1.75, *p* = .09) or consistent trials (*t*(41) < 1).

We next assessed the relationship between right IFG activity and susceptibility to misinformation on the final memory test (accuracy on misleading trials) across individuals (collapsing across warning conditions). A significant positive correlation was observed between activity in right IFG and memory accuracy on misleading trials (*r* = 0.42, *p* = .005, 95% CI [0.14, 0.64]; Fig. 2C). Importantly, there was not a relationship between this frontal activity and accuracy on consistent trials (*p =* 0.25) or neutral trials (*p =* .45). Furthermore, we confirmed a similar pattern was observed between right IFG activity and the rate of misinformation selection on misleading trials *(r* = −0.29*, p* = .06, 95% CI [−0.54, 0.01]). This pattern is expected given the strong negative correlation between accuracy on misleading trials and the rate of misinformation selection on misleading trials described above.

Exploratory follow-up analysis with a more lenient cluster threshold of *k* = 10 (see *Materials and* *methods*) revealed seven additional activations (Supplemental Table 1A; Supplemental Fig. 1). Of note, a cluster was observed in one of our a priori predicted regions, anterior cingulate cortex, which displayed a similar pattern to the right IFG cluster across trial types. Specifically, there was a significant interaction between condition and trial type (*F*(2, 82) = 4.89, *p* = .010,* n*^*2*^_*p*_ = .107) and significant effect of condition (*F*(1, 41) = 4.205, *p* = .047,* n*^*2*^_*p*_ = .093) which was driven by an increase in activity specifically on misleading trials in the prewarning group (*t* (41) = 3.37, *p* = .002, *d* = 1.03). Like activity in right IFG, activity in the ACC also showed a positive relationship with memory accuracy on misleading trials, although it did not reach statistical significance (*r* = .252, *p* = .103, 95% CI [−0.052, 0.513].

##### ROI analysis

Given our a priori predictions about potential group differences in the MTL, we performed an ROI analysis of activity in the hippocampus and parahippocampal cortex during misleading trails. No group differences were observed in left hippocampus (*t*(41) = 0.23, *p* = 0.82) or right hippocampus (*t*(41) = −0.85, *p* = 0.40). Similarly, no group differences were observed in left parahippocampal cortex (*t*(41) = 1.03, *p* = 0.31) or right parahippocampal cortex (*t*(41) = −0.36, *p* = .73). No group differences were observed in any of these regions on consistent trials (*p*s > .26) or neutral trials (*p*s > .29).

#### Attenuated Processing Hypothesis

**Fig. 3:**
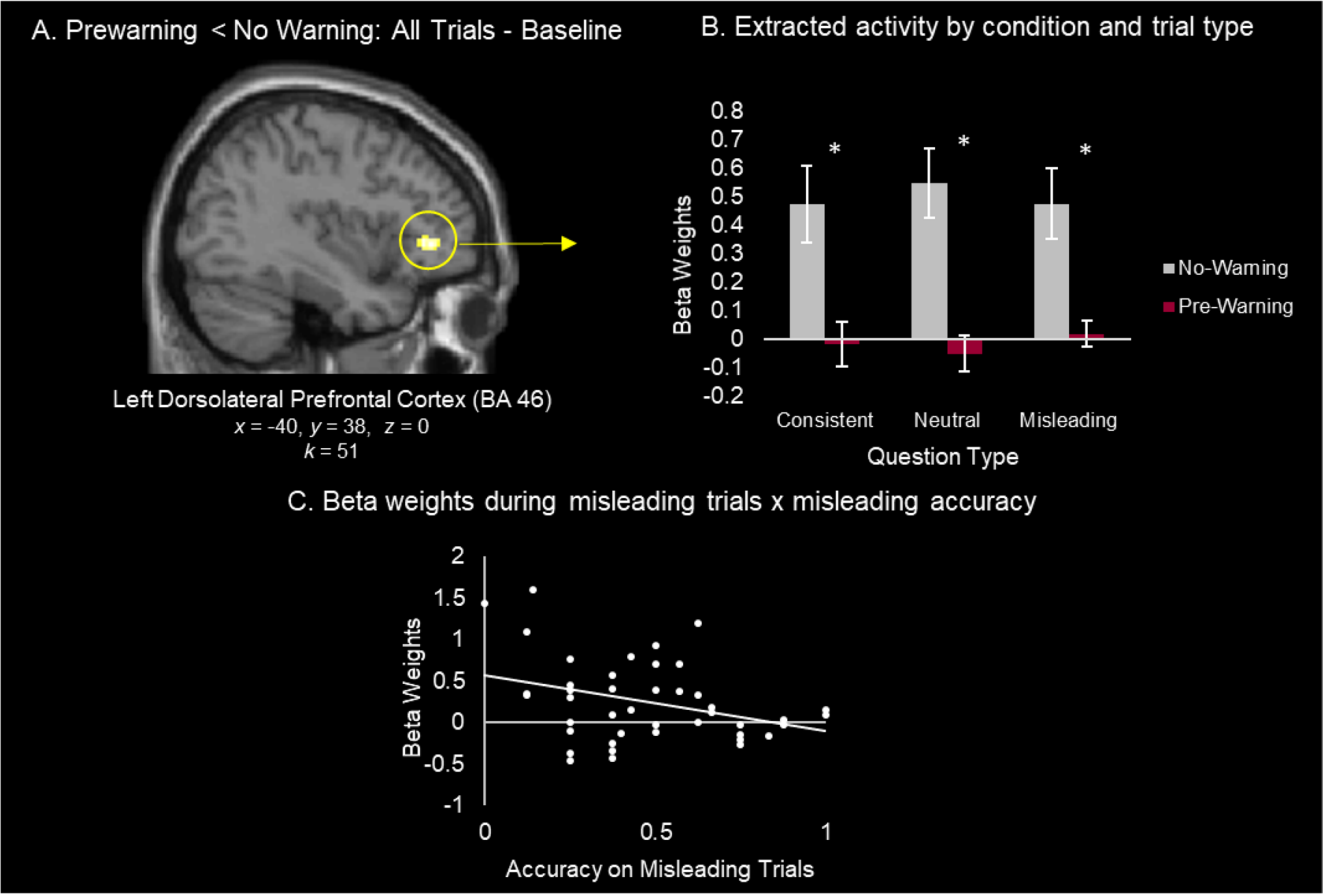
Whole Brain: Prewarning < No Warning. (**A**) Frontal activation was revealed by the contrast of prewarnings < no warning for All Trials – Baseline. (**B**) Extracted beta weights from the region are plotted by trial type and condition. (**C**) Collapsing across warning condition, extracted beta weights are plotted in relation to accuracy on misleading trials on the final memory test

We next explored whether prewarnings might have a more global influence on neural activity during the Post-Event Information phase across all trials (e.g., attenuated processing) by comparing activity in the no warning versus the prewarning groups across all trials. Activity was reduced in the prewarning compared to the no warning group across all trials in a single cluster falling in the left dorsolateral prefrontal cortex (dlPFC; BA 46; *x* = −40, *y* = 38 , *z* = 0; Fig. 3A). There was a significant main effect of condition (prewarnings, no warning; (*F*(1, 41) = 17.68, *p* < .001, *n*^*2*^_*p*_ = .30) and no main effect of trial type (*F* < 1) nor interaction between condition and trial type (*F* < 1). A post hoc independent samples *t*-test revealed that activity was significantly greater for prewarned compared to unwarned participants on consistent (*t* (41) = 3.12, *p* = .003, *d* = .95), misleading (*t* (41) = 3.40, *p* = .002, *d* = 1.04), and neutral (*t* (41) = 4.33, *p* < .001, *d* = 1.32) trials (Fig. 3B).

We next assessed whether the magnitude of this activity in left dlPFC region during misleading trials was related to memory accuracy on the final memory test across participants (collapsing across warning conditions). Indeed, a significant correlation was observed (*r* = −0.32, *p* = .036, 95% CI [−0.57, −0.02]; Fig. 3C), whereby reduced activity in left dlPFC was positively associated with protection from misinformation (i.e., memory accuracy on misleading trials). We again confirmed a similar pattern between left dlPFC activity and the rate of misinformation selection on misleading trials (*r* = 0.36, *p* = .02, 95% CI [0.07, 0.60]). This pattern is expected given the strong negative correlation between accuracy on misleading trials and the rate of misinformation selection on misleading trials described above.

An exploratory follow-up analysis with a more lenient cluster threshold of *k* = 10 (see *Methods*) revealed two additional clusters (Supplemental Table 1B; Supplemental Fig. 2). Given our hypotheses, a cluster in auditory cortex (BA 22, *k* = 31) was of particular interest. This region displayed a significant main effect of condition (*F* (1, 41) = 12.443, *p* < .001, *n*^*2*^_*p*_ = .23) but no main effect of trial type (*F* < 1) nor was there an interaction between condition and trial type (*F* < 1). Post hoc *t*-tests revealed that activity was significantly reduced for prewarned compared with postwarned participants across consistent (*t* (41) = 3.48, *p* < .001, d = 1.07), misleading (*t* (41) = 3.35, *p* = .002, *d* = 1.02), and neutral (*t* (41) = 3.16, *p* = .003, *d* = .96) trials.

Given that attenuated processing could also be evident by increased activity in the default mode network (Baym & Gonsalves, 2010), we also performed a whole-brain analysis to identify regions where activity might be *greater* in the prewarning compared to the no warning group across all trials. No clusters survived correction for multiple comparisons (*p* < .005, *k* = 38), but an exploratory follow-up analysis with a more lenient cluster threshold of k = 10 (see *Materials and methods*) revealed clusters in visual areas (BA 18, BA 37) as well as parietal regions (BA 7, BA 40) (Supplemental Table 1C; Supplemental Fig. 3). Condition (prewarning, no warning) by trial type (consistent, neutral, misleading) mixed ANOVAs revealed a significant effect of warning (*p*s < .01) with no interaction (*p*s > .37) or effect of trial type (*p*s > .11).

## Discussion

Previous research has demonstrated that warnings about the threat of misinformation can alter the manner in which that information is retrieved, thereby improving the accuracy of memory reports. The present study is the first, to our knowledge, to identify the neural mechanisms by which warnings impact encoding-related processes during initial exposure to misinformation. We had two primary hypotheses, both of which were supported by the data. First, based on previous literature (Okado & Stark, 2005), we hypothesized that prewarnings could protect memory from misinformation by increasing neural activity in regions associated with source encoding during exposure to misleading information that conflicts with an original event. Congruent with our hypothesis, we found that compared to unwarned participants, warned participants demonstrated increased activity in lateral prefrontal regions (BA 44) associated with source encoding (Cansino et al., 2002) as well as anterior cingulate regions associated with the detection of conflict (Carter & Van Veen, 2007). Importantly, a positive relationship was observed between activity in these regions and protection from misinformation on the final memory test (subsequent memory accuracy on misleading trials). These results align with findings in the fMRI literature that conflict-triggered attention mediated by the ACC and lateral PFC can enhance memory encoding and predict subsequent retrieval accuracy (Krebs et al., 2015) as well as findings in the misinformation literature that noticing discrepancies or changes between an original event and post-event information can lead to improved memory accuracy for the original event (Tousignant et al., 1986; Putnam et al., 2017).

Second, we hypothesized that prewarnings could protect memory from misinformation by encouraging shallower processing of the Post-Event Information at the more global level (“attenuated processing hypothesis”; Baym & Gonsalves, 2010). In support of this hypothesis, we found that participants who received a prewarning had reduced activity in the left dlPFC (BA 46) across all trials in the Post-Event Information phase. Such global decreases in left dlPFC activity across trials may be a marker of overall reduced attention to, and processing of, the verbal narrative in prewarned participants as compared to unwarned participants (for a review, see Kim, 2011). In support of this interpretation, exploratory analysis revealed that activity in auditory cortex (BA 22) was also reduced in the prewarning group across trials during the post-event auditory narrative. Importantly, activity in left dlPFC was negatively correlated with subsequent memory performance on misleading trials, suggesting that reduced activity in this region may play a role in protecting memory from misinformation. While the above-discussed results support the attenuated processing hypothesis, it is important to note that we did not find any difference in memory performance between warning groups on consistent or neutral trials on the Final Memory Test, which suggests that the prewarned participants still processed the Post-Event Information to some degree. It is also worth noting that we did not observe increased activity in the default mode network in the prewarning group, which could have provided additional evidence in support of the attenuated processing hypothesis (Baym & Gonsalves, 2010).

As described earlier, a prominent explanation for the misinformation effect is source misattribution at the time of memory retrieval. A number of theoretical models have been proposed to explain the underlying mechanism by which such source misattribution errors occur. The multiple trace model (Nadel & Moscovitch, 1997; Shao et al., 2023) predicts that representations of the Witnessed Event and Post-Event Information would exist independently, and misinformation errors occur when the wrong trace is accessed/selected during the Final Memory Test. Additionally, in a recent study, Bulevich et al. (2022) suggested that warnings may promote greater contextual discrimination between two highly accessible sources of information. Memory opposition tests (McClosky & Zaragoza, 1985) and modified opposition tests (Eakin et al., 2003) used in misinformation paradigms have consistently demonstrated that original information and the source of that information remains accessible, and the cause of misinformation selection, or source misattributions, may stem from memory trace confusion. Most recently, Shao & colleagues (Shao et al., 2023) employed a misinformation paradigm in which participants viewed visual scenes (Witnessed Event), were exposed to written summaries of what they saw but with some details altered (Post-Event Information), and then completed a Final Memory Test. In testing this theory, Shao and colleagues found that the hippocampus maintained distinct traces of both the Witnessed Event and the Post-Event Information during encoding of the Post Event Information. Furthermore, they found that misinformation errors on the Final Memory Test were more likely to occur when the hippocampal trace for the Post-Event Information was stronger than the hippocampal trace for the Witnessed Event. Similarly suggesting that multiple traces exist at the time of memory retrieval, Karanian & colleagues (Karanian et al., 2020) demonstrated that warnings about the reliability of Post-Event Information encouraged reinstatement of the Witnessed Event during the Final Memory Test, thereby leading to fewer misinformation errors. Specifically, compared with unwarned participants, warned participants were more likely to reinstate the Witnessed Event, as indicated by greater visual activity during memory retrieval, and were less likely to reinstate the Post-Event Information, as indicated by less auditory activity during memory retrieval. These results suggest that external manipulations, such as warnings, can facilitate selection of the proper source/trace at the time of memory retrieval, which provides support for the multiple trace theory.

In the present study, a primary finding was that prewarnings enhanced activity in ventrolateral frontal regions associated with source encoding during the Post-Event Information phase and that the magnitude of this activity was positively associated with performance on the final memory test, such that greater activity during misinformation exposure was associated with fewer source misattribution errors. These results are consistent with the idea that a distinct memory trace is maintained for Post-Event Information. Our current findings further suggest that that if prewarnings make source encoding stronger, then this could lead to a better ability to disambiguate between sources of information on the final memory test. It is worth mentioning that the above-discussed results refute other models, including the altered trace theory (Loftus et al., 1978), which suggests the Post-Event Information trace overwrites/alters the Witnessed Event thereby making some of the original accurate details inaccessible at the time of the Final Memory Test.

At first pass, it may seem puzzling that warnings appeared to enhance activity associated with source encoding on misleading trials as well as attenuate activity associated with sensory processing and attention more globally across trials. However, one possibility is that both mechanisms work in parallel to ultimately reduce source misattributions during the Final Memory Test. Specifically, whereas warnings may reduce processing of the Post-Event Information at the global level, warnings also could increase the detection of discrepancies between Post-Event Information and memory for the Witnessed Event (i.e., on misleading trials). In turn, this could enhance item-related source encoding whereby misleading details are tagged with contextual information so that they can be disambiguated from original event details in memory. This idea is consistent with hypothesized *recall-to-reject* processes that have been found to facilitate the rejection of test foils that are similar to studied stimuli (Gronlund & Ratcliff, 1989; Clark & Gronlund, 1996). *Recall-to-reject* processes operate by detecting traces that are retrieved from memory that are possible targets. This process uses information about a recalled item specifically to reject another. Although speculative, this could suggest that warnings have multiple effects on encoding-related processing during exposure to misinformation. In support of this possibility, a secondary analysis using stepwise linear regression found that activity in right IFG on misleading trials significantly predicted memory performance (*R*^2^ = .19, *F* (1, 41) = 9.70, *p* = .003) and that adding activity in left dlPFC to the second step of the model improved its capacity to account for variance in susceptibility to misinformation (memory accuracy on misleading trials; *R*^2^ change = .10, *F* (1, 40) = 5.39, *p* = .025).

The results of our exploratory whole brain analyses also revealed interesting patterns that might provide insight into the underlying mechanisms by which warnings protect memory from misinformation. In addition to reducing activity in auditory processing areas (BA 22) across all trials of the Post-Event Information phase, warnings also *increased* activity in visual processing regions (BA 18, BA 37). Given that the trials presented during the Post Event Information were purely auditory, it is plausible that such increases in visual activity in prewarned participants reflects sensory reinstatement of the Witnessed Event, which was presented visually (Wheeler et al., 2000; Karanian et al., 2020). Such reinstatement of the Witnessed Event may reflect increased source monitoring during the Post-Event Information, which could ultimately allow participants to better avoid source misattribution at the time of memory retrieval. While speculative, support for this possibility comes from the finding that the magnitude of this visual activity positively correlated with later memory performance on misleading trials. Accordingly, one post hoc explanation is that the memory benefit observed in the prewarned group during the Final Memory Test is not primarily driven by attenuated processing of Post-Event Information, but instead by enhanced encoding processes that involve actively comparing details from the Post-Event Information to details from the original Witnessed Event. This is an avenue for further research.

There are several factors that make the present study distinct from the previous fMRI literature. First, to our knowledge, this is the first study to investigate the impacts of warning on neural activity during exposure to misinformation. While previous studies have investigated neural activity during exposure to misinformation and its relationship to subsequent memory (Okado & Stark, 2005; Baym & Gonsalves, 2010; Shao et al., 2023), no study to date has investigated the effects of warnings on such encoding-related activity. As is typical in misinformation paradigms, participants can only be exposed to limited amount of misinformation during the Post-Event Information phase. Despite the small number of misleading trials in the present study, we were able to identify effects of prewarning and relate such activity to subsequent memory performance on misleading trials. Future warning studies could attempt to increase the number of misleading trials during Post-Event Information phase to conduct a standard subsequent memory analysis in which misleading trials could be parsed into subsequently remembered and subsequently forgotten categories and the effects of warning could also be examined. It also is important to note that the present paradigm employed a repeated testing procedure such that participants engaged in memory retrieval before exposure to misinformation. A number of studies on repeated testing suggests that engaging in retrieval affects susceptibility to subsequently encountered misinformation. It should be underscored that this paradigm difference makes it difficult to directly compare the results of the present study and previous fMRI misinformation studies (Okado & Stark, 2005; Baym & Gonsalves, 2010; Shao et al., 2023). Future studies would benefit from manipulating the number of retrieval attempts to better understand how repeated retrieval in the context of warnings affects neural processing of misinformation.

## Conclusions

The present study assessed the effects of prewarnings on neural processing during exposure to Post-Event Information. Consistent with previous literature, we find that prewarnings encouraged enhanced processing of source information during Post-Event Information, and such enhancement was associated with fewer misinformation errors on the final memory test. This work highlights the potential benefit of employing prewarnings in real-world contexts to protect eyewitness memory reports from the misinformation effect.

### Supplementary Information

Below is the link to the electronic supplementary material.

Supplementary file1 (DOCX 285 KB)

## Data Availability

Data will be made available on OSF.
