# Supplementary material for: Warning before misinformation exposure modulates memory encoding
Source: Cogn Affect Behav Neurosci. 2024 Mar 19;24(3):440–52. doi: 10.3758/s13415-024-01183-y (PMC11078839; doi:10.3758/s13415-024-01183-y)
Supplement: Supplementary file 1 — Supplementary file1 (DOCX 285 KB) [file 13415_2024_1183_MOESM1_ESM.docx]

**Supplemental Table 1.** Contrasts were conducted at an individual voxel threshold of *p* < .005 with a minimum cluster size of *k* = 10. Beta weights from each cluster were extracted and a condition (prewarnings, no warning) x trial type (consistent, neutral, misleading) ANOVAs were conducted for each region and reported in the Cond., Trial, and Int. columns. Pearson correlations were conducted to assess the relationship between beta weight magnitudes on misleading trials and memory performance on misleading trials on the final memory test.

*Region BA x y z t k Cond. Trial Int. Corr.*

1. **Prewarning > No warning: Misleading – Arrows**

Inferior Frontal Gyrus 44 48 18 8 3.90 61 .003 .603 .024 .005

Frontal Eye Fields 8 14 28 48 4.12 23 .110 .738 .008 .078

Visual Area 18 26 -86 -4 3.90 23 .002 .295 .622 .014

Fusiform 37 44 -54 8 3.73 23 .001 .389 .332 .022

Anterior PFC 10 -18 50 14 3.47 16 .032 .422 .067 .007

Frontal Eye Fields 8 30 4 38 3.37 13 .005 .524 .380 <.001

Anterior Cingulate 24 0 24 18 3.64 12 .047 .255 .010 .103

Posterior Parietal 7 10 -52 48 2.99 10 .182 .363 .015 .006

1. **Prewarning < No Warning: All Trials – Arrows**

Dorsolateral PFC 46 -40 38 0 3.75 51 <.001 .944 .522 .036 #

Superior Temporal 22 48 -34 4 3.70 31 .001 .712 .968 .809 #

Insula 13 -40 -2 -6 3.25 14 .002 .383 .365 .490 #

1. **Prewarning > No Warning: All Trials – Arrows**

Fusiform 37 42 -52 8 3.69 23 <.001 .334 .539 .007

Visual Area 18 26 -86 -4 3.48 19 .002 .383 .365 .010

Parietal 7 26 -66 50 3.18 13 <.001 .839 .644 .159

Supramarginal Gyrus 40 -36 -40 48 3.15 12 .008 .106 .764 .169

*Note: Whole-brain contrasts were conducted at p < .005, k = 10; BA = Brodmann Area. Cond. Reports the p-value of the main effect of condition, Trial reports the p-value of the main effect of trial type, and Int. reports the p-values of the interaction between condition and trial type. Corr refers to the p-value for the correlation between extracted beta weights from that region during encoding of misleading trials and performance on misleading trials on the final memory test. A # next to the p-value indicates a negative correlation, whereas no # indicates a positive correlation.*

**Supplemental Figure 1.** *Prewarning > No Warning: Misleading – Baseline*. Mean beta weights from each region identified by this contrast are plotted by condition (prewarnings, no warning) and trial type (consistent, neutral, misleading). Statistics are included in Supplemental Table 1.

**
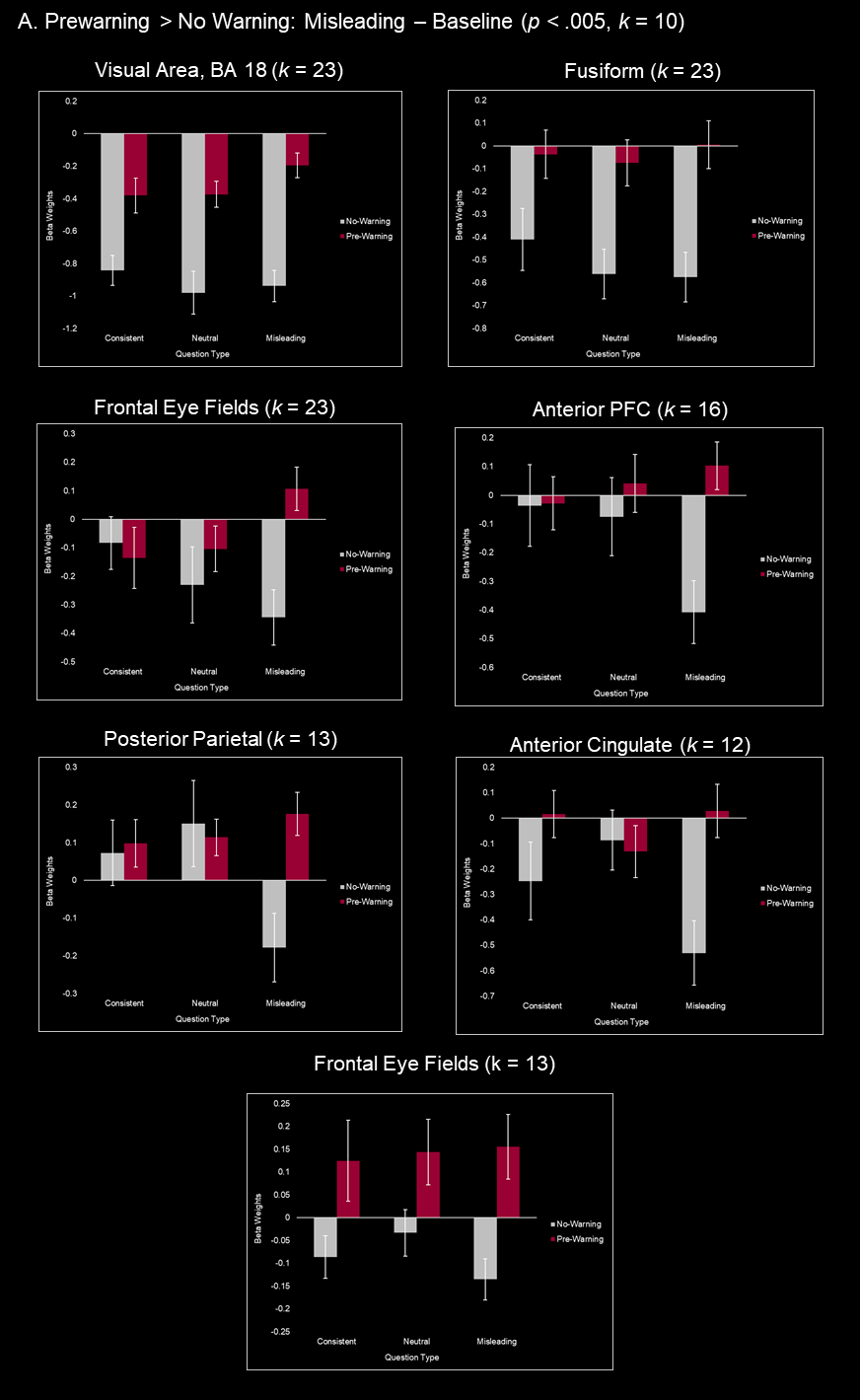
**

**Supplemental Figure 2.** *Prewarning < No Warning: All Trials – Baseline*. Mean beta weights from each region identified by this contrast are plotted by condition (prewarnings, no warning) and trial type (consistent, neutral, misleading). Statistics are included in Supplemental Table 1.

**
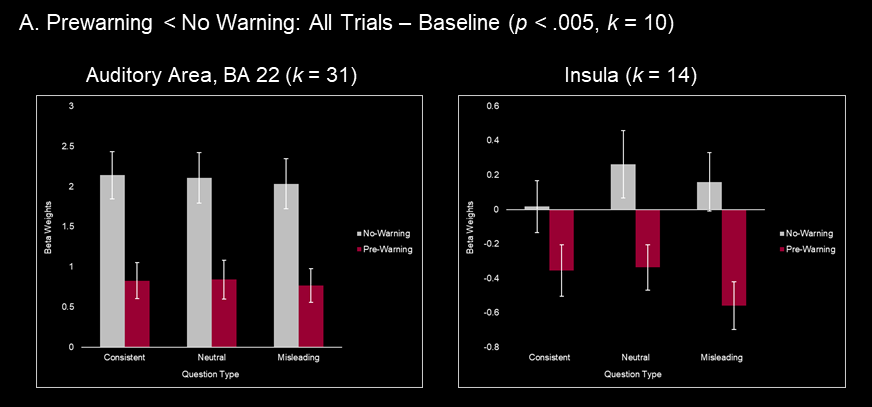
**

**Supplemental Figure 3.** *Prewarning > No Warning: All Trials – Baseline*. Mean beta weights from each region identified by this contrast are plotted by condition (prewarnings, no warning) and trial type (consistent, neutral, misleading). Statistics are included in Supplemental Table 1.

**
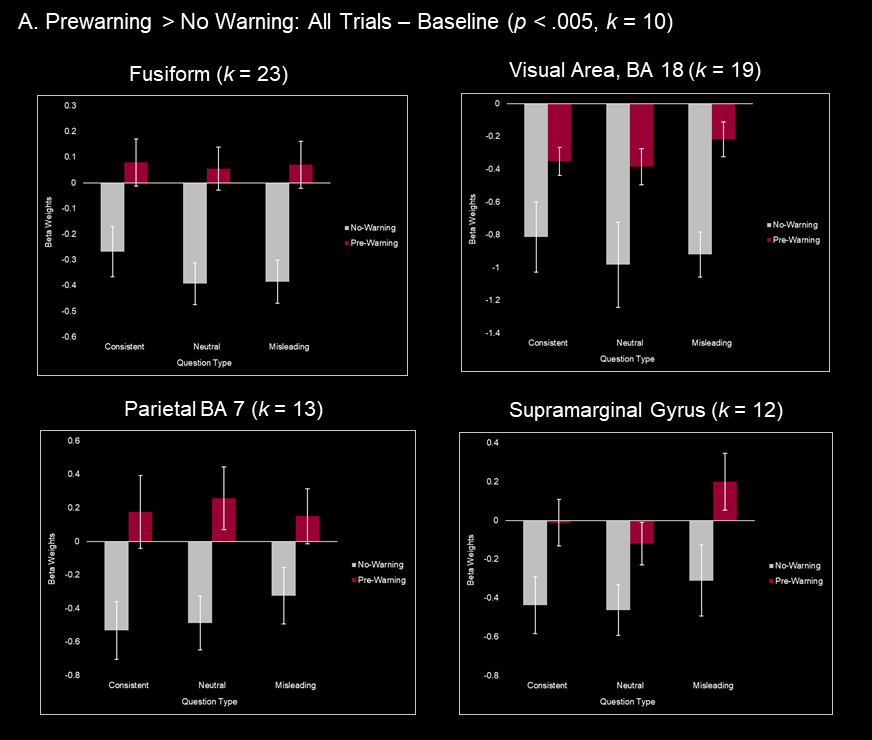
**

**Appendix A**

**No Warning:**

- **Prior to the audio narrative:** "You will now hear an audio narrative of the video you just watched. During the narrative, please keep your eyes fixed on the cross on the screen.”
- **After the audio narrative:** "You will now answer a series of questions relating to the video you watched at the beginning of this experiment. Please answer each question to the best of your ability. If you do not know the answer, make your best guess. All questions must be answered. Select your answer using the numbers and then wait for the screen to advance. After each question, please rate your confidence in your answer on the scale from 1, complete guess to 4, high confidence.”

**Pre-warning**

- **Prior to paying the audio narrative:** “You will have to answer questions regarding the video you previously watched for a second time. We will play a narrative of that video; however, we are uncertain as to the source of the narrative. Therefore, we were unable to verify the accuracy of the narrative. As such, base your answers only on what you saw in the video, and not on what you hear in the narrative. During the narrative, please keep your eyes fixed on the cross on the screen.”
- **After playing the audio narrative:** "You will now answer a series of questions relating to the video you watched at the beginning of this experiment. Please answer each question to the best of your ability. If you do not know the answer, make your best guess. All questions must be answered. Select your answer using the numbers and then wait for the screen to advance. After each question, please rate your confidence in your answer on a scale from 1, complete guess to 4, high confidence.”

**Post-warning**

- **Prior to playing the audio narrative:** "You will now hear an audio narrative of the video you just watched. During the narrative, please keep your eyes fixed on the cross on the screen.”
- **After playing the audio narrative:** “You will have to answer questions regarding the video you previously watched for a second time. We just played a narrative of that video; however, we are uncertain as to the source of the narrative. Therefore, we were unable to verify the accuracy of the narrative. As such, base your answer only on what you saw in the video, and not on what you heard in the narrative. Please answer each question to the best of your ability. If you do not know the answer, make your best guess. All questions must be answered. Select your answer using the numbers and then wait for the screen to advance. After each question, please rate your confidence in your answer on a scale from 1, complete guess to 4, high confidence.”

**Declarations**

**Funding**

This material is based upon work supported by the National Science Foundation under Grant 1728764 and NIH under Shared Instrumentation Grant S10OD021569.

**Conflicts of interest/Competing interests**

Not applicable

**Ethics approval**

Appropriate ethics approval was obtained from Tufts University and the Massachusetts Institute of Technology.

**Consent to participate (include appropriate statements)**

Informed consent was obtained from all individual participants included in the study.

**Consent for publication (include appropriate statements)**

Not applicable

**Availability of data and materials**

Data will be made available on OSF.

**Code availability**

Not applicable

**Authors' contributions (Optional)**

J.M.K., A.K.T., and E.R. designed research, J.M.K. A.K.T. and E.R. performed research, J.M.K. and E.R. analyzed data, and J.M.K., A.K.T., and E.R. wrote the paper.
